# A Case of Pseudoappendicitis Caused by Campylobacter Enteritis Diagnosed by Gram Staining and Direct Microscopic Investigation of Stool Specimen

**DOI:** 10.7759/cureus.33980

**Published:** 2023-01-19

**Authors:** Hidetaka Tabata, Yuta Horinishi, Chiaki Sano, Ryuichi Ohta

**Affiliations:** 1 Gastroenterology, Fuchu Hospital, Osaka, JPN; 2 Community Care, Unnan City Hospital, Unnan, JPN; 3 Community Medicine Management, Shimane University Faculty of Medicine, Izumo, JPN

**Keywords:** general medicine, rural hospital, stool test, gram stain, pseudoappendicitis, campylobacter infection

## Abstract

Campylobacter infection may progress to a systemic infection through the intestinal tract. In many cases, symptoms are within the self-limiting range and do not require multidisciplinary treatment. In contrast, systemic infections in younger patients may be more severe and require hospitalization. Many differential diagnoses are considered when Campylobacter infection presents with severe abdominal pain, and the initial diagnosis may be difficult. We encountered a patient with Campylobacter infection who presented with acute-onset fever and general malaise. We diagnosed the case in a resource-poor setting by performing Gram staining of stool samples and fecal microscopy. This case suggests that a diagnosis of Campylobacter pseudoappendicitis can be made efficiently by combining various stool tests rather than waiting for culture results.

## Introduction

Campylobacter infection is a major cause of acute diarrhea. The main pathogens, *Campylobacter jejuni* and *Campylobacter coli*, inhabit the intestinal tracts of various domestic animals. The route of infection is often unknown, but it can be caused by oral ingestion of raw or undercooked meat or food secondarily contaminated with raw meat [1]. After an incubation period of approximately three days, infections are established mainly in the colon and rectum [2]. The disease is found worldwide, regardless of the climate. In the past decade, the rate of Campylobacter infection has increased globally [3]. The initial symptoms of Campylobacter infections are generally abdominal pain, diarrhea, and bloody stool. However, in approximately one-third of cases, chills, generalized pain, dizziness, and high fever with delirium precede these gastrointestinal symptoms. Colonic inflammation can spread to the right iliac fossa and result in pseudoappendicitis. Definitive diagnostic techniques include stool culture and nucleic acid amplification testing, including reverse transcription polymerase chain reaction. Fecal microscopy and Gram staining are also available but are not performed effectively [2]. In this case, a young woman with a chief complaint of fever was quickly diagnosed using fecal microscopy and Gram staining. Through a discussion of this case, we present a diagnostic method that enables the timely diagnosis and treatment of Campylobacter infection without waiting for culture results in general clinical practice.

## Case presentation

A 35-year-old woman presented to our hospital with a chief complaint of fever and diarrhea. She had a fever of 38.4°C and malaise for three days before presenting to our hospital. After two days, she had nausea and acute intermittent abdominal pain in the lower part with no alleviating factors; therefore, she visited our emergency room and was diagnosed with gastroenteritis. However, the patient’s symptoms did not improve. After several watery diarrhea, vomiting water and food for one day, and inability to consume water, the patient again presented to our emergency room. She had ingested an uncooked liver six days before her visit. There were no people with the same symptoms as her. She had never experienced the same symptoms before. She had a history of irregular menstrual periods and was taking a combination of norgestrel and Ethinyl estradiol tablets.

On arrival, her blood pressure was 130/80 mmHg, pulse rate was 88 beats/min, respiratory rate was 20 breaths/min, temperature was 38.8°C, and oxygen saturation was 97%. Physical examination revealed no abnormalities in the head, neck, or chest. The abdomen was flat and soft, with mild tenderness in the right lower abdomen and no obvious signs of peritoneal irritation. Blood samples showed mildly elevated white blood cell and platelet counts (Table 1).

**Table 1:** Initial laboratory data of the patient

Biomarkers	Level	Reference
Blood count		
White blood cells	10.9	3.5-9.1×10^3^/μL
Hemoglobin	13.7	11.3-15.2 g/dL
Mean corpuscular volume	91.1	79.0-100 fl
Platelets	30.8	13.0-36.9×10^4^/μL
Biochemistry		
Albumin	3.8	3.8-5.3 g/dL
Total bilirubin	0.3	0.2-1.2 mg/dL
Aspartate aminotransferase	22	8-38 IU/L
Alanine aminotransferase	19	4-43 IU/L
γ-Glutamyl transpeptidase	35	< 48 IU/L
Lactate dehydrogenase	195	121-245 U/L
Blood urea nitrogen	4.0	8-20 mg/dL
Creatinine	0.59	0.40-1.10 mg/dL
Serum Na	134	135-150 mEq/L
Serum K	3.5	3.5-5.3 mEq/L
Serum Cl	101	98-110 mEq/L
Serum Ca	9.5	8.8-10.2 mg/dL

Urinalysis showed pyuria. Abdominal ultrasonography revealed ascites in the lower right abdomen. Therefore, computed tomography (CT) of the abdominopelvic region was performed. CT showed no obvious appendiceal enlargement but the presence of wall thickening from the ileum to the ascending colon and several lymphadenopathies in the ileocecal region.

**Figure 1:**
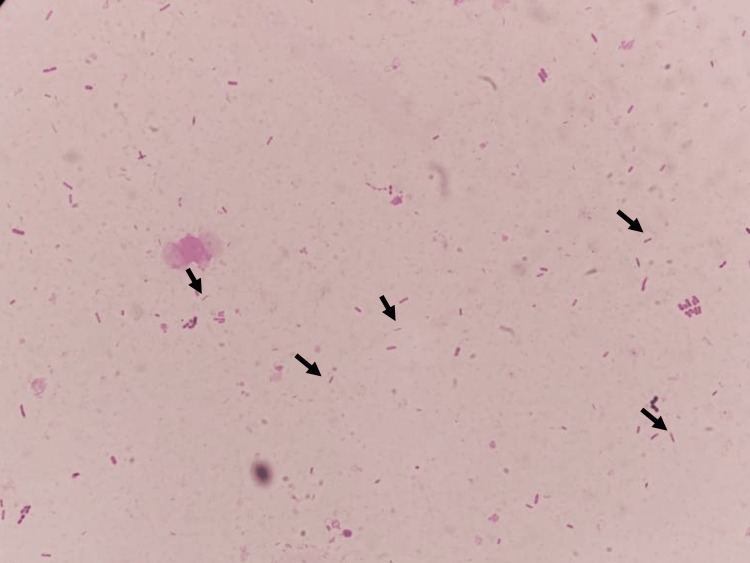
Microscopic examination of a stool showing corkscrew-like fast-moving bacteria (black arrows)

Considering the possibility of infectious colitis, Gram staining of the stool was performed, which revealed a Gram-negative spiral bacillus with a gull-wing (Figure 1).

To further confirm the presence of spiral bacilli, fecal microscopy was performed. Fecal microscopy revealed corkscrew-like moving bodies with monoflagellated flagella (Video 1).

**Video 1:** Fecal microscopy showing corkscrew moving bacteria with monoflagellated flagella

The patient was diagnosed with ileitis and colitis caused by *C. jejuni*. After three days of treatment with fasting and intravenous fluids, the patient’s abdominal pain was resolved, and she was discharged.

## Discussion

In the present case, a young woman developed a fever, abdominal pain, and diarrhea. A fecal microscopic examination showed the presence of corkscrew-shaped organisms, and Gram staining showed the presence of Gram-negative helical bacteria with gull wings, which could facilitate the diagnosis of pseudoappendicitis with Campylobacter infection in addition to imaging tests. In this case, Gram-stained Gram-negative spiral bacteria with gull wings allowed rapid diagnosis of Campylobacter enterocolitis.

The ileocecal region is the focus of inflammation in yersinia infection. Differential diagnosis of pseudoappendicitis and appendicitis is critical in yersinia enterocolitis. In our case, fever, diarrhea, vomiting, and an increased white blood cell count were observed in the lower right abdomen. This presentation could be confused with appendicitis due to inflammation of the ileocecal region. This condition is called pseudoappendicitis syndrome and is caused by different bacteria [2]. *Campylobacter *is a motile, spore-free, curved, Gram-negative rod-shaped bacterium. The name is derived from the Greek word for a curved rod, which refers to its vibrio-like morphology [1]. Their helical shape and long polar flagella allow them to move quickly and survive within the mucosal layer of the gastrointestinal tract [1]. Campylobacter infection usually begins in the jejunum and ileum and progresses distally to affect the cecum and colon [2]. In the case of pseudoappendicitis, as in the present case, the fecal examination and imaging tests can identify the causative microorganism, which can then be used to differentiate it from appendicitis and promptly treat the patient.

In Campylobacter enteritis, systemic symptoms may precede gastrointestinal symptoms, and the diagnosis may take time. Although the pathogenesis of Campylobacter enteritis remains unclear, it has been reported that *C. jejuni* activates mitogen-activated protein kinase (MAP kinase), an inflammation-related signal, in intestinal epithelial cells, causing the secretion of the inflammatory cytokine interleukin-8 (IL-8) [4]. Systemic inflammation occurs before the spread of inflammation in the intestinal mucosa [4]. This systemic inflammation stimulates intravascular monocytes and macrophages, which promote the secretion of inflammatory cytokines, such as IL-6 and tumor necrosis factor, and systemic symptoms, such as fever and fatigue, may precede systemic inflammation [4]. In the present case, unlike most cases of enteritis, fever preceded gastrointestinal symptoms, raising the possibility of Campylobacter enteritis.

Furthermore, when systemic symptoms precede the inflammatory symptoms, enteritis may become more severe and require hospitalization and treatment [5]. Moreover, tenderness in the ileocecal region may be observed, even in the presence of fever and malaise preceding gastrointestinal symptoms [6,7]. By quickly confirming the abdominal findings and performing ultrasonography or CT to determine inflammation in the ileocecal region early in the course of symptoms, a diagnosis of pseudoappendicitis can be made [8]. Prompt antimicrobial agents should be administered in severe cases. In close examination and treatment of patients with fever, appropriate abdominal examinations are important for diagnosis. In rural contexts, a lack of medical resources demands that rural general physicians must be able to perform precise physical examinations to promote effective diagnoses, as in this case [9]. Thus, rural general physicians should be educated to systematically examine patients and diagnose them in precise differential diagnoses with minimal tests such as Gram staining, direct microscopy, and ultrasonography or CT [10].

## Conclusions

Campylobacter infection may cause pseudoappendicitis, and a thorough abdominal examination should be performed, even in the absence of gastrointestinal symptoms in patients with fever. In addition, early diagnosis and treatment of Campylobacter enteritis may be possible by performing Gram staining of feces and microscopic examination of fresh stool specimens. Rural general physicians must be able to perform precise tests that can be quickly completed.
